# Diabetic Muscular Atrophy: Molecular Mechanisms and Promising Therapies

**DOI:** 10.3389/fendo.2022.917113

**Published:** 2022-06-30

**Authors:** Yuntian Shen, Ming Li, Kexin Wang, Guangdong Qi, Hua Liu, Wei Wang, Yanan Ji, Mengyuan Chang, Chunyan Deng, Feng Xu, Mi Shen, Hualin Sun

**Affiliations:** ^1^ Key Laboratory of Neuroregeneration of Jiangsu and Ministry of Education, Co-Innovation Center of Neuroregeneration, National Medical Products Administration (NMPA) Key Laboratory for Research and Evaluation of Tissue Engineering Technology Products, Jiangsu Clinical Medicine Center of Tissue Engineering and Nerve Injury Repair, Nantong University, Nantong, China; ^2^ Department of Laboratory Medicine, Department of Endocrinology, Binhai County People’s Hospital affiliated to Kangda College of Nanjing Medical University, Yancheng, China; ^3^ Department of Orthopedics, Haian Hospital of Traditional Chinese Medicine, Nantong, China; ^4^ Department of Endocrinology, Affiliated Hospital 2 of Nantong University and First People’s Hospital of Nantong City, Nantong, China; ^5^ Nanjing Institute of Tissue Engineering and Regenerative Medicine Technology, Nanjing, China

**Keywords:** diabetes mellitus, muscle atrophy, molecular mechanism, treatment, inflammation

## Abstract

Diabetes mellitus (DM) is a typical chronic disease that can be divided into 2 types, dependent on insulin deficiency or insulin resistance. Incidences of diabetic complications gradually increase as the disease progresses. Studies in diabetes complications have mostly focused on kidney and cardiovascular diseases, as well as neuropathy. However, DM can also cause skeletal muscle atrophy. Diabetic muscular atrophy is an unrecognized diabetic complication that can lead to quadriplegia in severe cases, seriously impacting patients’ quality of life. In this review, we first identify the main molecular mechanisms of muscle atrophy from the aspects of protein degradation and synthesis signaling pathways. Then, we discuss the molecular regulatory mechanisms of diabetic muscular atrophy, and outline potential drugs and treatments in terms of insulin resistance, insulin deficiency, inflammation, oxidative stress, glucocorticoids, and other factors. It is worth noting that inflammation and oxidative stress are closely related to insulin resistance and insulin deficiency in diabetic muscular atrophy. Regulating inflammation and oxidative stress may represent another very important way to treat diabetic muscular atrophy, in addition to controlling insulin signaling. Understanding the molecular regulatory mechanism of diabetic muscular atrophy could help to reveal new treatment strategies.

## Introduction

Diabetes mellitus (DM) is a common chronic metabolic disease. There are two main subtypes of endocrine cells in pancreatic islets: β cells and α cells. Islet β cells are involved in the production of insulin, while islet α cells are responsible for the secretion of glucagon. These cell types work together to maintain an appropriate blood glucose level in the human body. DM can be mainly divided into type 1 diabetes mellitus (T1DM) and type 2 diabetes mellitus (T2DM). T1DM accounts for less than 10% of all instances of DM, with T2DM accounting for more than 90% (1). DM is mainly induced by two causes: the impairment of insulin secretion (insulin deficiency) and insulin resistance (2). The former results from islet β cell dysfunction, while the latter refers to the loss of insulin-mediated cellular glucose uptake in DM patients. Increased insulin levels reduce the affinity of insulin receptors, meaning that cells gradually become insensitive to insulin.

DM is often accompanied by secondary complications that involve multiple organs, such as the eyes, kidneys, heart, and brain, as well as skeletal muscle (3). To date, relevant studies have mainly discussed the risks of cardiovascular disease, blindness, and renal failure in DM patients (4). In addition, DM also induces a shift in muscle fiber phenotype from slow-twitch to fast-twitch, which can lead to skeletal muscle atrophy, energy metabolism disorders, and muscle weakness (1, 4). Muscle atrophy is caused by an imbalance between the synthesis and degradation of protein (5). Maintaining muscle homeostasis is crucial for preserving the body’s integrity and function. Muscle atrophy has also been associated with a variety of diseases, and can lead to a poor quality of life. Diabetic muscular atrophy is considered to be a DM complication; it is characterized by proximal lower extremity muscle weakness, atrophy, pain, sensory disturbances, and even quadriplegia in severe cases (6). Research into the molecular mechanism of diabetic muscular atrophy and its treatment strategies could aid the development of effective treatments and improve prognoses. To date, however, little research has been conducted in this regard.

Muscle atrophy is closely related to two major protein degradation pathways, the ubiquitin-proteasome system (UPS) and the autophagy-lysosome pathway (ALP). It is also related to the protein synthesis pathways, such as the insulin-like growth factor 1– phosphoinositide-3-kinase–Akt/protein kinase B–mammalian target of rapamycin (IGF1–PI3K–Akt/PKB–mTOR) pathway and IGF-1-AKT- Forkhead box O (FoxO) pathways (7–12). In T2DM, insulin resistance has been shown to inhibit protein synthesis by inhibiting the IGF-1-PI3K-AKT/PKB-mTOR pathway, and to activate the UPS and ALP through the IGF-1-AKT-FoxO signaling pathway, thereby promoting muscle atrophy. T1DM-induced muscle atrophy, meanwhile, is mediated by the FoxO-driven protein degradation pathway (13–15). In addition, oxidative stress damage, inflammatory response, and high levels of glucocorticoids (GCs) can all trigger muscle atrophy in DM patients (16, 17). Herein, we discuss the major molecular regulatory mechanisms of muscle atrophy and diabetic muscular atrophy, and describe potential drugs and treatments for the latter. Moreover, we provide new ideas and strategies to improve the prognosis of DM patients.

## Molecular Mechanisms of Skeletal Muscle Atrophy

### Protein Degradation Pathways

The UPS is the major hydrolysis system for cellular proteins; it is responsible for the degradation of misfolded or damaged cellular proteins in skeletal muscle (18, 19). Ubiquitin is a short protein comprising 76 amino acids; it can activate proteolysis in skeletal muscle (20). Most proteins are degraded by the 26S proteasome, through the modification of covalently attached polyubiquitin chains. The ubiquitination of proteins proceeds through a cascade of reactions that are catalyzed by a range of enzymes, including E1 ubiquitin-activating enzyme, E2 ubiquitin-conjugating enzyme, and E3 ubiquitin ligase (21). These tagged proteins are subsequently recognized by the 26S proteasome, which consists of a 20S core and two 19S regulatory complexes. These 19S regulatory complexes can recognize and bind to ubiquitinated proteins to undergo specific proteolysis (22). Cullin-RING E3 ubiquitin ligases (CRL) are the largest known class of ubiquitin ligases; they regulate a variety of cellular processes in skeletal muscle, including cellular proliferation, transcription, signal transduction, and development (23). Levels of the muscle-specific E3 ubiquitin ligases muscle RING-finger protein-1 (MuRF1) and muscle atrophy F-box (MAFbx)/Atrogin-1 have been shown to be significantly upregulated in atrophic skeletal muscle. These ligases are also known to be involved in the degradation of skeletal muscle proteins (24–26). Therefore, the UPS is one of the main mechanisms underlying muscle atrophy, and MuRF1 and MAFbx are two important regulators of muscle atrophy.

The ALP is a lysosomal degradation pathway that is widespread in eukaryotic cells; it is involved in the degradation and elimination of damaged, degenerated, aged, or dysfunctional organelles (27). The ALP is crucial for cell survival and repair, intracellular protein balance, and environmental homeostasis. Under starvation and other conditions, a double-layered membrane structure forms in cells; this structure then gradually extends to envelop undegraded proteins or cellular components, thereby forming autophagosomes. Autophagosomes are driven by cytoskeletal proteins to fuse with lysosomes, where their cargo components become degraded (28). Autophagy has dual functions. On the one hand, it can degrade damaged organelles and abnormal proteins, preventing them from accumulating in cells. On the other hand, autophagy overactivation can damage organelles, and thereby become toxic to cells. Studies have found that autophagy is crucial for maintaining skeletal muscle homeostasis, and that overactivated autophagy can promote muscle atrophy or autophagy impairment, leading to muscle degeneration (29, 30). Autophagy is activated under nutrient deprivation. Antimicrobial peptide-activated protein kinase (AMPK) inhibits the mTOR complex-1 (mTORC1) complex, which in turn inhibits protein synthesis, and can even lead to severe late-onset myopathy (31, 32). The studies demonstrate that autophagy plays an important role in skeletal muscle atrophy.

### Protein Synthesis Pathway

The IGF-1-PI3K-AKT-mTOR pathway is a positive regulator that is responsible for controlling protein synthesis (33). IGF-1 is a key growth factor that regulates skeletal muscle synthesis and catabolism; it also promotes the growth of muscle cells. The inactivation of muscle-specific IGF-1 receptors impairs muscle growth, triggering a reduction in the number, diameter, and cross-sectional area of muscle fibers (34). Conversely, the overexpression of muscle-specific IGF-1 receptors has been shown to lead to muscle hypertrophy (11). Then, IGF-1 can activate the PI3K-AKT signaling pathway and then stimulate mTOR activity (35). mTOR is assembled into different complexes such as mTORC1 and mTOR complex-2 (mTORC2). mTORC1 positively regulates the activation of its effector 70-kDa ribosomal protein S6 kinase (p70S6K) and negatively regulates the inhibitory of the translation initiation factor 4E-eukaryotic translation initiation factor 4E binding protein 1 (eIF4E-4EBP1) complex. This leads to increased protein translation and synthesis, which subsequently promotes muscle growth (36, 37).

In addition to IGF-1’s regulatory role in muscle growth and protein synthesis, the FoxO family also plays an important role in the pathophysiological process of skeletal muscle development. The FoxO family has three subtypes, FoxO1, FoxO3, and FoxO4, which control a series of atrophy-related genes in skeletal muscle, including MAFbx and MuRF1. The dephosphorylation of FoxO members can up-regulate MAFbx and MuRF1, thereby accelerating protein degradation and subsequently inducing muscle atrophy (5). FoxO’s transcription factor is the downstream target of the PKB/AKT pathway. AKT can phosphorylate all members of FoxO, with the resulting phosphorylated FoxO members being exported from the nucleus to the cytoplasm. This inhibits their transcriptional activity, and eventually represses muscle atrophy (38). Although AKT can inhibit the UPS and ALP (5), AKT remains inactive in the absence of growth factors. FoxO members will translocate to the nucleus and induce the transcription of the target genes of UPS and ALP (9), thereby initiating protein degradation.

## Molecular Mechanism of Diabetic Muscular Atrophy

**Figure 1:**
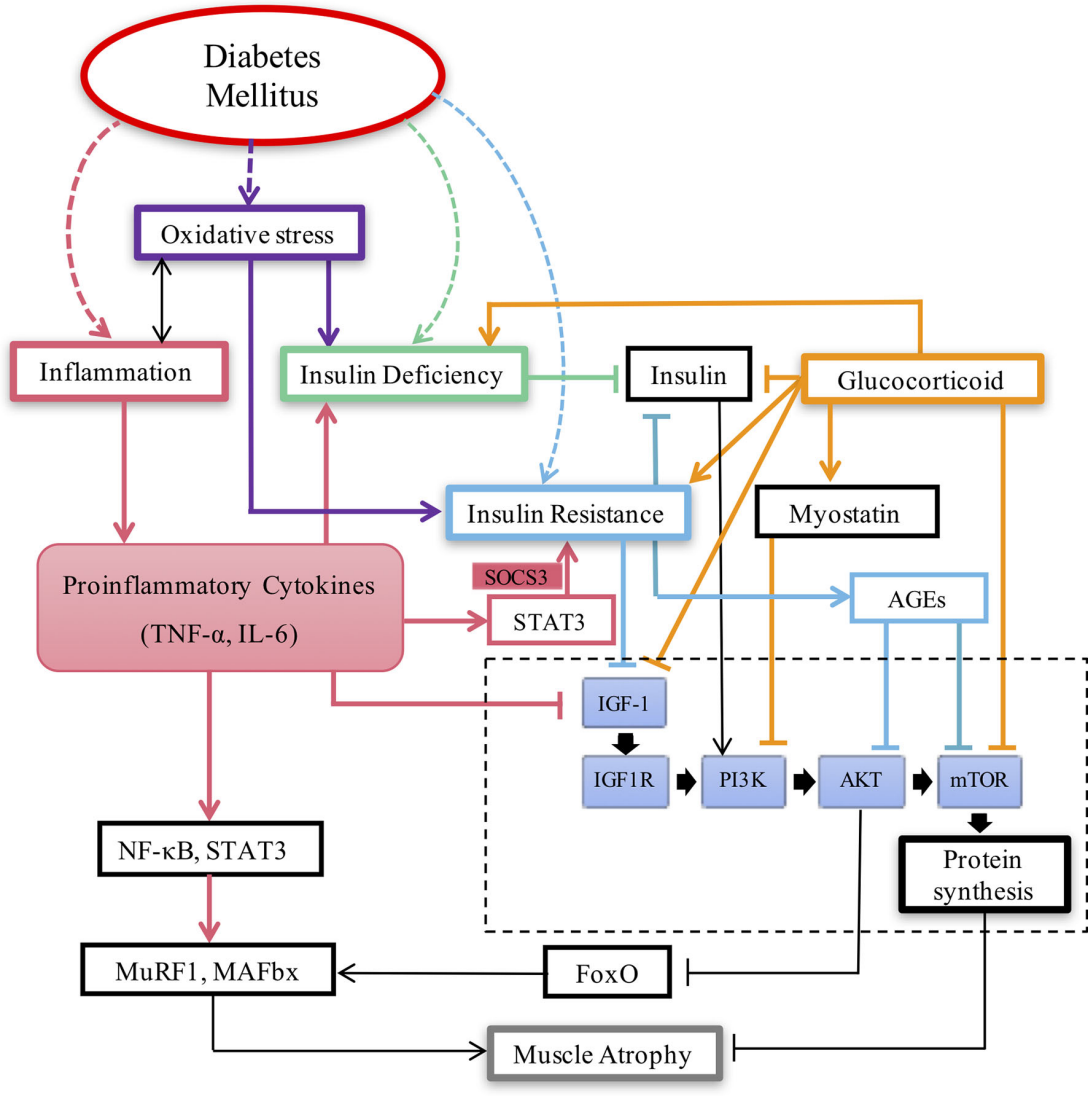
Key pathways involved in Diabetic muscular atrophy.

The molecular mechanism of diabetic muscular atrophy is very complicated. The current mainstream view is that diabetic muscular atrophy is closely related to insulin resistance, insulin deficiency, inflammation, oxidative stress, glucocorticoids and so on (**Figure 1**).

### Insulin Resistance

Insulin-stimulated glucose uptake is crucial for muscle contraction. Insulin is a powerful synthetic signal that significantly stimulates muscle protein synthesis (39). Classical insulin signaling pathways and anabolic stimuli, including PI3K, 3-phosphoinositide-dependent kinase 1 (PDK1), AKT, mTOR, and p70S6K, activate protein synthesis and thereby promote muscle growth (40, 41). DM-induced insulin dysfunction inhibits glucose uptake in skeletal muscle, thereby disturbing muscle contraction (42). Under normal conditions, the intracellular insulin signaling cascade activates the mTOR pathway and inhibits autophagy (including the lysosomal degradation of proteins and organelles). However, such effects are deactivated in the presence of insulin resistance, which may accelerate muscle loss in patients with DM (43). In addition, sarcopenia is a complication of T2DM that is characterized by the progressive loss of skeletal muscle mass and function (44, 45). Sarcopenia is related to the weakness and geriatric syndrome of the human body; it can impact quality of life for elderly patients, and can even lead to death in severe cases (46). Due to low muscle mass, sarcopenia may lead to insulin resistance through altered glucose disposal (47).

When insulin resistance occurs, insulin or IGF-1 signaling is inhibited. This results in the inactivation of the PI3K/AKT pathway, which in turn inhibits mTOR activity and reduces protein synthesis; these effects may ultimately lead to muscle loss in T2DM patients (48). In addition, insulin resistance leads to elevated systemic glucose levels, with glucose being able to react with proteins or lipids to generate advanced glycation end products (AGEs) (49). AGEs play an important role in the pathogenesis of chronic diabetic complications. Moreover, the accumulation of AGEs is a potential cause of muscle loss and muscle weakness in T2DM patients (50). Receptor for advanced glycation end products (RAGE) is a transmembrane signaling receptor that is associated with diabetic renal and vascular complications. AGEs can induce muscle atrophy or myogenesis impairment through the RAGE-mediated, AMPK-induced downregulation of AKT signaling (51). Furthermore, AGEs have been shown to modulate muscle anabolic signaling by inhibiting the mTORC1 signaling pathway (50). Overall, insulin resistance can inactivate the IGF-1-PI3K-AKT-mTOR protein synthesis pathway, thereby reducing protein synthesis and ultimately inducing skeletal muscle atrophy.

### Insulin Deficiency

Patients with T1DM exhibit a reduced repair capacity regarding their skeletal muscle satellite cells, as well as skeletal muscle dysfunction. Both of abnormal phenotypes are associated with insulin deficiency, which causes the rates of protein degradation exceed that of protein synthesis (52). Under normal conditions, insulin receptor (IR) and IGF-1 receptor (IGF-1R) can act through the PI3K/AKT pathway on a variety of cellular functions. For example, during glucose uptake and protein synthesis, the activation of AKT in response to insulin or IGF-1 can phosphorylate FoxO transcription factors, thereby inhibiting their transcriptional activity (4). Insulin-deficient diabetes, or a loss of insulin/IGF-1 action in muscle, reduces complex I-driven mitochondrial respiration and supercomplex assembly through the FoxO-mediated inhibition of complex I subunit (53). These effects impact mitochondrial function and induce skeletal muscle atrophy. In a biopsy from patients with T1DM who experienced insulin deficiency for 8 h, transcripts of the UPS and ALP were found to be increased, indicating that muscle atrophy in T1DM is induced by FoxO-driven protein degradation. Therefore, blocking this pathway may protect against diabetic complications (4). In short, when insulin is deficient, the transcriptional activity of FoxO becomes enhanced, which in turn promotes the expression of muscle atrophy-related genes (such as MAFbx and MuRF1) and causes muscle atrophy.

### Inflammation

Interleukin-6 (IL-6) is a pro-inflammatory cytokine that has catabolic effects on muscle (54). Patients with T2DM have elevated levels of C-reactive protein, IL-1β, and IL-6 (55). In T1DM, the ability of skeletal muscle regeneration is impaired due to dysfunction of satellite cells. The transient elevation of IL-6 leads to the proliferation of satellite cells, while the slow elevation of IL-6 impairs satellite cell function (56). Therefore, chronically elevated IL-6 levels may be responsible for satellite cell dysfunction in DM. In addition, hyperglycemia can promote the release of inflammatory mediators, such as IL-6. It can also stimulate macrophages, some other innate immune cells, and activate some apoptosis-related signaling pathways, such as the Fas/FasL signaling pathway (57, 58). Such stimulation can lead to islet β cell dysfunction, subsequently causing insulin deficiency (2). In addition, IL-6 can also induce insulin resistance by reducing insulin sensitivity, or by affecting lipid metabolism (59, 60). Insulin acts by binding to IR. Tumor necrosis factor alpha (TNF-α), which is a pro-inflammatory cytokine, can destroy the tyrosine phosphorylation activation of IR and IR substrate (IRS) in the insulin signaling cascade, thereby leading to insulin resistance (60). In addition, TNF-α can also reduce the glucose uptake and utilization of skeletal muscle and adipocytes by reducing the expression of glucose transporter 4 (GLUT4). This leads to insulin resistance (61), thereby promoting muscle atrophy.

Furthermore, the systemic inflammatory responses caused by obesity and long-term overnutrition not only induce typical insulin resistance in T2DM patients, but also reduce protein synthesis in muscle, thereby promoting UPS- and ALP-mediated protein degradation and facilitating the progression of muscle atrophy (62). Signal transducer and activator of transcription 3 (STAT3) can be activated by pro-inflammatory cytokines (such as IL-6); this weakens protein synthesis-related signaling pathways in muscle (63–66). Nuclear factor-κB (NF-κB) is an important transcriptional regulator that can induce the expression of various genes by activating stimulatory factors (viruses, tumor necrosis factor, and B cell activating factor) (67, 68). Additionally, NF-κB can increase the degradation of specific muscle proteins by increasing the expression of MuRF1 (69–71). NF-κB and STAT3 signaling pathways function as inflammatory pathways that can be significantly activated by increases in pro-inflammatory cytokines (i.e., TNF-α) and non-esterified fatty acids. This then increases the expression of MuRF1, thereby activating the UPS (72, 73). IL-6-activated STAT3 can induce insulin resistance through suppressors of cytokine signaling 3 (SOCS3); it can then inhibit the PI3K-AKT pathway, which reduces protein synthesis (74, 75) and increases myostatin transcription (72). These effects are significant as they can improve the progression of muscle atrophy. NF-κB and STAT3 may be involved in muscle atrophy in T2DM patients (62). In addition, IL-6 can also induce muscle atrophy *via* the regulation of IGF-1 (37, 74). Overall, inflammation can reduce protein synthesis through the inhibition of IGF-1 and the induction of insulin resistance, activate the UPS through the FoxO family and their downstream E3 ubiquitin ligases, and promote the expression of atrophy-related genes (possibly through the NF-κB and STAT3 pathways). Thus, it can eventually lead to muscle atrophy.

### Oxidative Stress

Excess production of reactive oxygen species (ROS) in the human body can induce oxidative stress, which damages lipids, proteins, and deoxyribonucleic acid (DNA) (76). In addition, obesity and hyperglycemia can also lead to oxidative stress (77–79). The high metabolic capacity of skeletal muscle makes it susceptible to oxidative stress injuries (37). Oxidative stress inhibits the AKT-mTOR pathway and its downstream targets, which subsequently inhibits protein synthesis and promotes muscle atrophy (80, 81). In addition, islet β cells are particularly sensitive to ROS due to their inherent antioxidant enzymes at low levels. ROS can directly damage β cells and promote apoptosis. Moreover, they can indirectly regulate the insulin signaling pathway and inhibit the function of β cells, leading to the occurrence of DM (82, 83). ROS is an important mediator for activating pro-inflammatory signaling pathways (84, 85). A chronic inflammatory environment is also conducive to producing free radicals, such as ROS. This can aggravate β-cell damage, thereby generating a positive feedback loop in which further harmful cytokines are then secreted, triggering further damage to β cells (86). Oxidative stress can induce insulin deficiency, and can produce large quantities of ROS to hinder insulin signaling transduction, thereby triggering insulin resistance (87). Eventually, this can contribute to the development of skeletal muscle atrophy. Therefore, oxidative stress injury plays an key role in the process of skeletal muscle atrophy.

### GCs

GC is a hypoglycemic hormone that promotes gluconeogenesis and glycogen breakdown, thereby counteracting the action of insulin and increasing blood glucose levels (88). GC signaling significantly contributes to muscle atrophy in DM (89). In addition, when GC binds to glucocorticoid receptor (GR), it inhibits AKT, GLUT4, and IR signals, and then induces insulin resistance (90). In T1DM caused by insulin deficiency, when insulin deficiency coexists with GCs in muscle, GR can compete with IRS1 to bind PI3K subunits P110 and p85. This results in a decrease in the phosphorylation levels of IRS, PI3K, and AKT, eventually leading to muscle atrophy (89, 90). GCs mainly cause muscle atrophy by increasing protein breakdown through the UPS and ALP, and by reducing protein synthesis *via* the inhibition of the IGF-1-PI3K-AKT-mTOR and mTOR/p70S6k pathways (91–93). In addition, GCs upregulate the production of myostatin, which reduce protein synthesis *via* the AKT-mTOR pathway (94). GCs can also induce muscle atrophy by binding to their receptors, thereby interfering with the insulin/IGF-1 signaling pathway and stimulating the transcription of dystrophin. Moreover, GRs can synergize with FoxO1 to induce MuRF1, thereby accelerating muscle atrophy (95). GRs also upregulate the expression of regulated in development and DNA damage responses 1 (REDD1) and Kruppel like factor 15 (KLF15). KLF15 is a member of the KLF transcription factor family; it can regulate muscle catabolism by regulating MAFbx and MuRF1 (96). The expression of KLF15 has been found to be up-regulated in the livers of diabetic mice, and hyperglycemia is known to up-regulate KLF15 protein, thereby accelerating skeletal muscle atrophy (97). REDD1 is a stress-responsive protein that inhibits the targets of mTOR in mTOR1 (98). The inhibition of mTOR can upregulate KLF15, which would increase the expression of atrophy-related genes, thereby triggering atrophy (96, 99). Overall, GCs participate in skeletal muscle atrophy through multiple pathways.

### Other Factors

Glucose can stimulate the degradation of WW domain-containing E3 ubiquitin protein ligase 1 (WWP1) through the proteasome pathway. The downregulation of WWP1 inhibits the ubiquitin-dependent degradation of KLF15 and then up-regulates KLF15 expression. This increases the expression of muscle atrophy-related genes, resulting in the loss of skeletal muscle mass (100). IGF-1 activates PDK1 to exert a core role in anabolic signaling. Subsequently, PDK1 activates the AKT/mTOR/p70S6k pathway (101), which further promotes protein synthesis. Moreover, it inhibits protein degradation by inhibiting the FoxO1 transcription factor (102). Increasing amounts of evidence have shown that microRNAs are involved in the regulation of skeletal muscle homeostasis. The overexpression of miR-193b in T1DM patients has been shown to reduce the expression of PDK1 and the phosphorylation of AKT, mTOR, p70S6k, and AMPK. In this way, it can inhibit protein synthesis and enhance the expression of MAFbx and MuRF1, thereby promoting proteolysis (103). To summarize, the mechanism that causes diabetic muscular atrophy is very complex; it warrants further in-depth study and exploration.

## Treatments and Therapeutic Drugs

To gain a systematic understanding of the impact of commonly used anti-diabetic drugs on muscle atrophy and discuss potential therapeutic drugs, we outline various studies on the drugs to treat diabetic muscular atrophy (**Table 1**).

**Table 1:** Treatments and therapeutic drugs for diabetic muscular atrophy.

Drugs	Function	Mechanism	Effect on muscle	References
Metformin	Targeted insulin resistance	Activate AMPK	Promote repair of skeletal muscle	(104, 105)
	Anti-inflammatory	Inhibit NF-κB		(106)
Thiazolidinedione	Targeted insulin resistance	Inhibition of protein hydrolysis and induction of PGC-1α to reduce the expression of atrophy-related genes	Attenuate the muscle wasting	(104)
Insulin	Targeted insulin deficiency	Promote the synthesis of protein; inhibit the decomposition of protein	Indirect benefit to skeletal muscle	(104)
Aspirin	Antioxidation	Reduce the production of ROS	Attenuate muscle wasting	(66)
	Anti-inflammatory	Inhibition of Janus Kinase (JAK)/STAT and NF-κB signaling pathway		(107)
	Targeted insulin resistance			
Omega-3 fatty acid	Antioxidation	Inhibit the production of ROS	Against Muscle atrophy	(108)
	Anti-inflammatory	Decreased Activation of NF-κB; stimulated MTORC1 signal		(109–111)
Vitamin D	Antioxidation	Get rid of ROS	Prevent and cure muscle atrophy	(112)
	Anti-inflammatory	The proinflammatory cascade reaction (NF-κB, TNF-α) was down-regulated, and the expression of FOXO1 was decreased		(112, 113)

### Targeting Insulin Resistance and Insulin Deficiency

The commonly used drugs for T2DM include metformin, glinides, thiazolidinediones, and peptidyl peptidase-4 inhibitors, all of which can improve insulin sensitivity. Metformin is a first-line drug for T2DM; it can eliminate the deleterious effects of DM on human bones. By activating AMPK, metformin can not only increase the translocation of glucose transporter 4 to the cell membrane, but can also contribute to skeletal muscle repair (104, 105). Glinides stimulate insulin secretion to reduce blood glucose level by closing the ATP-sensitive potassium channels (KATP) channel of islet β cells (114). However, repaglinide, which is a kind of glinide, can induce skeletal muscle atrophy and sarcopenia (104). Therefore, this drug may be not the ideal therapeutic drug of DM. Thiazolidinediones function as an important class of insulin sensitizers; they can not only improve insulin sensitivity to DM (115), but can also inhibit proteolytic pathways and stimulate mitochondrial biogenesis. Thiazolidinediones can partially induce the expression of peroxisome proliferator-activated receptor-gamma coactivator 1 alpha (PGC-1α), so as to reduce the expression of atrophy-related genes in muscles of patients with T2DM (104). Thus, they may be able to prevent diabetic muscular atrophy. Insulin remains the main treatment for T1DM; it can promote protein synthesis and inhibit protein breakdown (104). It should be noted that the dosages of these drugs need to be adjusted temporally according to a patient’s blood glucose levels. As mentioned above, insulin deficiency induces muscle protein degradation through the FoxO-dependent pathway. Therefore, blocking this pathway can prevent diabetic muscular atrophy, the complications of DM. Moreover, this could provide new insights into the prevention and treatment of diabetic muscular atrophy.

### Anti-Inflammation and Antioxidation

There is positive feedback between inflammation and oxidative stress. Inflammation could induce cellular oxidative stress and oxidative stress also could lead inflammation (12, 73, 116). Aspirin is a non-steroidal anti-inflammatory drug that has been shown to alleviate insulin resistance and hyperglycemia in patients with T2DM by inhibiting the Janus kinase (JAK)/STAT and NF-κB signaling pathways (117). At the same time, aspirin also has good antioxidant properties in denervation induced muscle atrophy, as evidenced by reduced reactive oxygen species (66). However, the long-term use of nonsteroidal anti-inflammatory drugs is not recommended due to their adverse side effects. Other anti-inflammatory and antioxidant supplements, such as omega-3 fatty acids and vitamin D, represent viable options for long-term, daily usage (50). Omega-3 fatty acids, such as eicosapentaenoic and docosahexaenoic acids, are ingested through one’s diet. It is well known that inflammation and oxidative stress could be restricted by omega-3 fatty acids. Omega-3 fatty acids can ameliorate inflammation, reduce proinflammatory cytokine, inhibits free radicals (ROS) (107). They can reduce NF-κB activation by blocking the activation of Toll-like receptor 4 (TLR4) signaling (induced by lipopolysaccharides or saturated fatty acids). This subsequently exerts an anti-inflammatory effect (108). Higher ratios of omega-3 to omega-6 will lead to lower production of pro-inflammatory mediators derived from omega-6 (109). Omega-3 has also been shown to stimulate mTORC1 signaling, and thus could be used to repress muscle atrophy (110). Vitamin D, a ROS scavenger, can also act as an anti-inflammatory and antioxidant mediator (111). Pro-inflammatory cascades (i.e., NF-κB and TNF-α) have been shown to be downregulated when vitamin D binds to receptors in macrophages and lymphocytes (112). Vitamin D has also been shown to regulate muscle growth. In a muscle-specific vitamin D receptor knockout model, insulin resistance was found to occur, accompanied by the increased expression and activity of FOXO1. In vitamin D-treated muscle cells prepared *in vitro*, reductions were observed in the expression, nuclear translocation, and activity of FOXO1 (113). Therefore, vitamin D regulates muscle growth in part through FOXO1 signaling; it could be thereby used to prevent skeletal muscle atrophy.

In addition to regulating muscle atrophy, metformin and thiazolidinediones have relatively strong anti-inflammatory activities (115, 118). Therefore, incidences of muscle atrophy-related complications (or the degree of muscle atrophy) decrease when these drugs are used to treat DM. Systemic inflammation can lead to insulin resistance, whereas exercises can reduce systemic inflammatory markers and improve systemic and local muscle inflammation. Consequently, muscle atrophy following exercise can be relieved by increasing protein synthesis *via* the upregulation of the IRS1-PI3K-AKT pathway, the downregulation of the UPS, and the strengthening of mTORC2 activation (62). Overall, therefore, anti-inflammatory and antioxidant therapy may constitute an important strategy for combatting diabetic muscular atrophy.

## Prospects

There is a growing understanding of the mechanism underlying DM-induced muscle atrophy. Insulin signaling plays a dominant role in controlling muscle size, and GC signaling significantly promotes muscle atrophy in patients with DM. In addition, inflammation, oxidative stress, and the activation of some signaling pathways can all also cause muscle atrophy in patients with DM. It is worth noting that inflammation is closely related to insulin resistance, insulin deficiency, and even oxidative stress regarding the mechanism of diabetic muscular atrophy. Therefore, regulating inflammation may represent another very important way to treat diabetic muscular atrophy, in addition to controlling insulin signaling. This could have important therapeutic implications for the treatment of diabetic muscular atrophy, and even DM. Oxidative stress is also an important cause of DM and its related complications, so inhibiting excessive ROS production is crucial for delaying the onset of DM. From the perspective of antioxidants, however, to date little progress has been made regarding the development of drugs for treating diabetic muscular atrophy. Protecting and restoring insulin function, reducing insulin resistance, and inhibiting inflammation through antioxidants all also represent viable research directions, as they could suppress diabetic muscular atrophy. Further in-depth research into the mechanism of diabetic muscular atrophy will contribute to the development of better treatments. A therapeutic method for diabetic muscular atrophy could potentially be developed through the co-regulation of insulin signaling, the GC signaling pathway, inflammation, and oxidative stress; this would be extremely valuable for improving the quality of life of patients with DM.

## Author Contributions

(I) Conception and design: HS, MS and FX. (II) Administrative support: HS, MS and FX. (III) Collection and assembly of data: YS, ML, KW, GQ, H, WW, YJ, MC, CD. (IV) Data analysis and interpretation: YS, ML, KW, GQ, HL. (V) Manuscript writing: All authors. (VI) Final approval of manuscript: All authors.

## Funding

This work was supported by the National Natural Science Foundation of China (Nos. 82072160, 81901933, 32130060), the Major Natural Science Research Projects in Universities of Jiangsu Province (No. 20KJA310012), the Natural Science Foundation of Jiangsu Province (Nos. BK20202013, BK20201209), the “QingLan Project” in Jiangsu Universities, the Priority Academic Program Development of Jiangsu Higher Education Institutions, Nantong Science and Technology Program (No. JC2021118).

## Conflict of Interest

The authors declare that the research was conducted in the absence of any commercial or financial relationships that could be construed as a potential conflict of interest.

## Publisher’s Note

All claims expressed in this article are solely those of the authors and do not necessarily represent those of their affiliated organizations, or those of the publisher, the editors and the reviewers. Any product that may be evaluated in this article, or claim that may be made by its manufacturer, is not guaranteed or endorsed by the publisher.
